# Structure determination of GPCRs: cryo-EM compared with X-ray crystallography

**DOI:** 10.1042/BST20210431

**Published:** 2021-09-28

**Authors:** Javier García-Nafría, Christopher G. Tate

**Affiliations:** 1 Institute for Biocomputation and Physics of Complex Systems (BIFI) and Laboratorio de Microscopías Avanzadas (LMA), University of Zaragoza, 50018 Zaragoza, Spain; 2 MRC Laboratory of Molecular Biology, Cambridge CB2 0QH, U.K.

**Keywords:** cryo-electron microscopy, crystallography, G-protein-coupled receptors

## Abstract

G protein-coupled receptors (GPCRs) are the largest single family of cell surface receptors encoded by the human genome and they play pivotal roles in co-ordinating cellular systems throughout the human body, making them ideal drug targets. Structural biology has played a key role in defining how receptors are activated and signal through G proteins and β-arrestins. The application of structure-based drug design (SBDD) is now yielding novel compounds targeting GPCRs. There is thus significant interest from both academia and the pharmaceutical industry in the structural biology of GPCRs as currently only about one quarter of human non-odorant receptors have had their structure determined. Initially, all the structures were determined by X-ray crystallography, but recent advances in electron cryo-microscopy (cryo-EM) now make GPCRs tractable targets for single-particle cryo-EM with comparable resolution to X-ray crystallography. So far this year, 78% of the 99 GPCR structures deposited in the PDB (Jan–Jul 2021) were determined by cryo-EM. Cryo-EM has also opened up new possibilities in GPCR structural biology, such as determining structures of GPCRs embedded in a lipid nanodisc and multiple GPCR conformations from a single preparation. However, X-ray crystallography still has a number of advantages, particularly in the speed of determining many structures of the same receptor bound to different ligands, an essential prerequisite for effective SBDD. We will discuss the relative merits of cryo-EM and X-ray crystallography for the structure determination of GPCRs and the future potential of both techniques.

## Introduction

**Figure 1.**
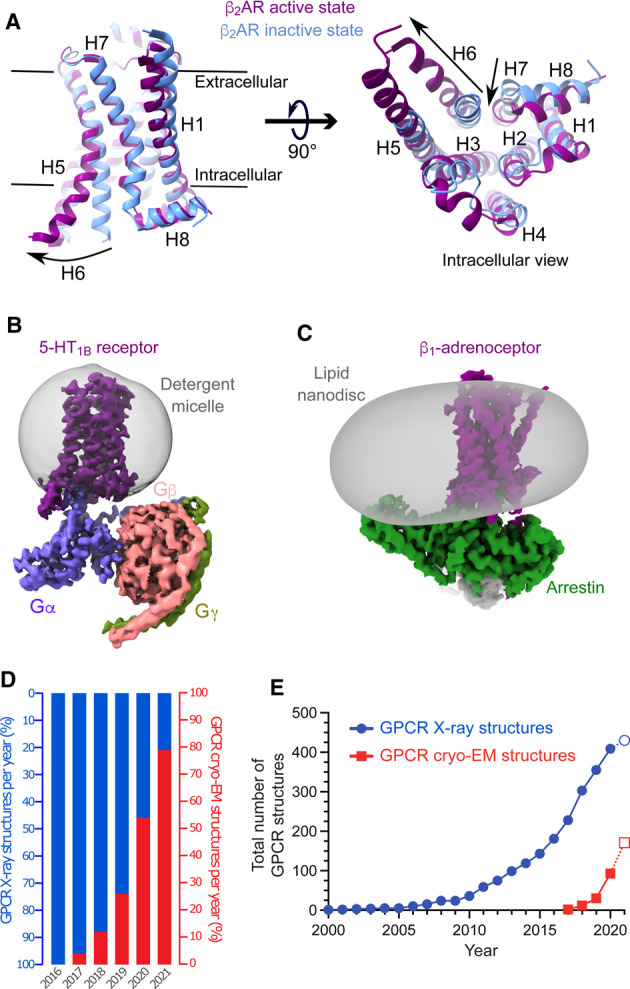
GPCR structures. (**A**) Cartoon of β_2_AR indicating the conformational change from the inactive state (blue; PDB ID 2RH1) [20] to the active state (purple; PDB ID 3SN6) [26] when coupled to a G protein (not shown). (**B**) Cryo-EM density for the serotonin 5-HT_1B_ receptor coupled to the heterotrimeric G protein G_o_ (PDB ID 6G79, EMDB-4358) [79]. The detergent used for purification of the receptor was decylmaltoside. (**C**) Cryo-EM density for the β_1_-adrenoceptor coupled to β-arrestin (PDB 6TKO, EMDB-10515) [9]. The detergent-purified receptor was reconstituted into a lipid nanodisc containing a bilayer of phosphatidylcholine and phosphatidylglycerol and has an external diameter of ∼13 nm. (**D**) Percentage of GPCR structures in the Protein Data Bank (PDB) determined by cryo-EM per year. (**E**) Cumulative number of GPCR structures determined by X-ray crystallography and cryo-EM, which includes multiple structures of the same receptor bound to different ligands, different intracellular binding partners and different species. Note that in panels **D** and **E** the data for 2021 includes only the first 7 months of the year.

G protein-coupled receptors (GPCRs) are the largest family of membrane proteins in humans with widespread distribution throughout the body [1,2], and are highly druggable with 34% of small molecule FDA-approved drugs targeting them [3]. GPCRs detect extracellular stimuli, such as hormones and neurotransmitters (agonists), and transduce this information into the cell through conformational changes, forming an active state of the GPCR that couples to heterotrimeric G proteins (consisting of an α, β and γ subunits) and β-arrestins (Figure 1B,C) [4,5]. Within heterotrimeric G proteins, the α-subunit is the main contributor to the specificity of coupling [6]. There are 16 genes in humans that encode α-subunits and they are classified into four families depending on their function and signalling cascade that they activate (G_s_, G_i/o_, G_q/11_ and G_12/13_) [7]. Given the high physiological and therapeutic value of GPCRs, considerable effort continues to be devoted to understanding their molecular pharmacology. Structures define how agonists (receptor activators) and antagonists (receptor inhibitors) bind to the receptor within the orthosteric binding pocket [2] and how agonist binding alters the receptor conformation from an inactive state (stabilised by antagonists) to an active state that allows G protein coupling (Figure 1A) [8]. Structures have also identified the specificity determinants for G protein [6] and β-arrestin coupling [9–12] and how coupling increases the affinity of ligands in the orthosteric binding pocket [13]. Understanding how the structure of the receptor changes during activation has also led to molecular insights into how some ligands bind in areas distant from the orthosteric binding site and either inhibit or facilitate receptor activation (negative and positive allosteric modulators, respectively) [14].

Structural biology provides crucial molecular details on all aspects of receptor biology. However, GPCRs are often unstable in the detergents required for their purification and crystallisation [15], which has made structural elucidation a challenge for decades. Until 2017, X-ray crystallography was the method of choice to obtain high-resolution structural information of GPCRs, and the structures of a variety of GPCRs were determined, mostly in an inactive state or intermediate state. The advent of high-resolution structure determination by electron cryo-microscopy (cryo-EM) [16] has triggered an explosion of membrane protein structures and, since 2019, the number of membrane protein structures determined by cryo-EM is higher than by X-ray crystallography [17]. Similarly, many GPCR structures have recently been determined in fully active states coupled to either a G protein or β-arrestin [18]. X-ray crystallography and cryo-EM now provide complimentary tools in understanding GPCR structure and function. Here, we provide a comparison of both approaches, their relative merits and future potential.

## Historical perspective on GPCR structural determination

In the 20th century, integral membrane proteins were challenging targets for X-ray crystallography because the small detergents necessary for crystal formation are denaturing and very often inactivated the protein during purification [15]. Thus, only a handful of very stable membrane proteins were crystallised. The first structure of a GPCR was that of rhodopsin in the year 2000, as it is naturally abundant and highly stable [19]. It took another seven years for the first ligand-activated receptor (β_2_-adrenoceptor; β_2_AR) to be crystallised [20,21], closely followed by the β_1_-adrenoceptor and the adenosine A_2A_ receptor the following year [22,23]. These and further advances in GPCR structural biology only occurred due to the development of complimentary tools, strategies and technical developments such as receptor thermostabilisation, receptor-T4 lysozyme fusion proteins, lipidic cubic phase (LCP) crystallisation, new detergents and microfocus synchrotron beamlines [24,25].

The first structures of GPCRs were mainly antagonist-bound inactive states or agonist-bound intermediate states. The fully active states of GPCRs are usually only attained when they are coupled to a G protein or β-arrestin (Figure 1A). Due to the flexibility of the complex and difficulties in forming crystal contacts, only two X-ray structures of a GPCR-G protein complex have been published [26,27]. Alternative strategies have also been developed to crystallise active states of GPCRs through the use of transducer mimetics, such as a C-terminal peptide of the α-subunit [28], conformation-specific nanobodies [29–31] and mini-G proteins [32,33]. These structures have been exceedingly informative in studying receptor conformations, but they are still very challenging to obtain and the structures did not provide the whole picture of how G proteins couple to receptors.

In contrast with X-ray crystallography, cryo-EM has proven to be an ideal technique for determining the structures of GPCR-G protein and GPCR-arrestin complexes. This has arisen from concerted developments over the past 10 years in new electron microscopes, direct electron detectors and image processing software, that together have transformed single-particle cryo-EM [34–36]. The first GPCR-G protein cryo-EM structure was determined in 2017 [37] and since then there has been an explosion of active state GPCR cryo-EM structures, which include previously intractable GPCR classes [38,39], complexes with different G proteins [6], β-arrestins [9,10,12] (Figure 1) and a G protein-coupled receptor kinase [40]. An inactive state cryo-EM structure of the CGRP receptor in complex with RAMP1 has also been determined, which is currently the smallest GPCR complex (73 kDa of ordered structure) solved by cryo-EM [41]. The ease of structure determination by cryo-EM has resulted in a rapid uptake of the technology by the GPCR community (Figure 1D,E) with 78% of 99 GPCR structures deposited in the PDB this year (Jan–Jul 2021) determined by cryo-EM. Currently, it is the technical requirements that dictate which technique is used for determining a structure of a GPCR, and these are discussed in the following sections.

## Which technique to use, X-ray crystallography or cryo-EM?

There are many interrelated factors that influence the choice of whether to use cryo-EM or X-ray crystallography to determine a structure of any given GPCR, not least the availability of relevant expertise. However, currently the GPCR itself dictates which route is most feasible and, if time is important, which is the fastest route. The size of the GPCR is the most critical factor. Currently, the lower size limit for the structure determination of a membrane protein is ∼60 kDa, so it is unrealistic to determine the structure of a 35–45 kDa GPCR in the inactive state by cryo-EM, although structure determination of large Class C receptor dimers (molecular weight ∼180 kDa) in the inactive state has been very successful [42–44]. In contrast with the inactive state, small GPCRs in the active state coupled to a heterotrimeric G protein (molecular weight of the complex ∼130 kDa) are well within the range of current cryo-EM technology. Clearly, binding an antibody F_ab_ fragment (molecular weight 50 kDa) or two camelid single chain antibodies (nanobodies; each ∼18 kDa in molecular weight) would be sufficient to increase the size of a GPCR in the inactive state to allow its structure determination by cryo-EM. The use of a F_ab_ to increase mass was also essential for the high-resolution structure determination of the β_1_AR-arrestin complex, in addition to its role in stabilising the active state of β-arrestin [9].

Stability is another key issue that can affect the tractability of GPCR structure determination by X-ray crystallography or cryo-EM. Crystal formation often takes days or even weeks and during this time the receptor has to maintain its structure. This can be enhanced by binding a high-affinity ligand to the receptor which will lock and stabilise the receptor in a preferred conformation [45]. Stability may also be enhanced by engineering the receptor through thermostabilising point mutations [46] or by binding an antibody [47]. In contrast with X-ray crystallography, the preparation of samples for cryo-EM can be performed in a matter of minutes after the sample has been purified, hence stability issues are generally better tackled by using cryo-EM.

Constraints on time and financial resources may also be important factors in deciding the best route to determine a GPCR structure, given that the fields of GPCR structural biology and drug discovery are highly competitive. Both X-ray crystallography and cryo-EM require purified protein in a stable monodisperse state to allow structure determination. However, extensive engineering of the GPCR is usually necessary to obtain well-diffracting crystals. For example, X-ray crystallographers will invariably remove any flexible regions of unstructured protein (N-terminus, C-terminus, intracellular loop) [48], remove post-translational modification sites (e.g. N-glycosylation, phosphorylation, palmitoylation etc) [48] and may make chimeras with well-folded proteins (e.g. T4 lysozyme, cytochrome b_562_) [49] to try and get well-diffracting crystals. In addition, if the receptor is unstable, thermostabilising mutations may have to be found and combined to make a sufficiently stable receptor [46]. Very often this is an iterative process, where poor crystals lead to further engineering until well-diffracting crystals can be obtained. This whole process may take years, and sometimes well-diffracting crystals are never obtained. In contrast, cryo-EM can be performed on receptors that contain unstructured regions and variable amounts of post-translational modifications, so sometimes it is possible to determine the structure of the native wild type receptor [38]. Receptor engineering may only be required to improve poor expression or if proteolysis of unstructured regions results in the loss of purification tags.

Cryo-EM is also more tolerant than X-ray crystallography in terms of sample quality and quantity, as small amounts of purified protein (∼100 µg), with some degree of receptor heterogeneity and the presence of contaminants can still allow a cryo-EM structure to be determined, but would invariably prevent the formation of good crystals. This is because processing of cryo-EM images can separate molecules of receptor with the same conformation and use these to determine a structure. Thus, out of 5–10 million particles in a typical cryo-EM dataset, only ∼100 000–300 000 particles may be used to form the final cryo-EM map. In contrast, in a GPCR crystal every molecule needs to be precisely aligned with its identical neighbour to be able to generate a diffraction pattern suitable to determine an electron density map. In a single thin GPCR crystal ∼10–20 µm long there are about a billion identical molecules, which is ∼10 000 times more than is required for a cryo-EM structure.

Another aspect of the time constraint issue is if multiple structures of the same receptor are required, such as when SBDD is being used to develop novel therapeutics [18]. Once a receptor has been crystallised and its structure determined, it can often be very fast to determine new structures bound to different ligands as the crystals can grow under similar conditions and molecular replacement can be used to rapidly determine the structure from the X-ray diffraction data. In some cases, it is even possible to soak crystals of GPCRs and determine multiple structures from one crystallisation experiment and one trip to collect diffraction data [50]. In contrast, with cryo-EM there is no equivalent to molecular replacement and each structure takes a similar time to determine, which is often days to weeks compared with a few hours for molecular replacement in X-ray crystallography.

## Resolution of structures

Historically, cryo-EM has yielded structures of lower resolution compared with X-ray crystallography. However, the dramatic developments in single-particle cryo-EM have resulted in a ‘resolution revolution’ [16] with recent structures of model proteins reaching atomic resolution (1.2 Å) [51,52]. The improvement in resolution has also been observed for GPCR complexes. In 2019 with the first dozen GPCR-G protein complexes solved [53] the average resolution was 3.76 Å with the best being 3.3 Å. In comparison, in the first six months of 2021, 48 GPCR-G protein cryo-EM structures were determined (21 unique receptors) with an average resolution of 3.11 Å (highest resolution, 1.95 Å) [54] and 16 structures with resolutions below 3 Å. The resolution of these structures is comparable to standard X-ray crystallography and has been achieved through further developments in cryo-EM technology [55].

In cryo-EM structures, density maps may show large variations in resolution throughout the receptor, with the core of the receptor often having the highest resolution and the loop regions the lowest resolution. This reflects the flexibility of many loop regions compared with the receptor core where side chains are packed together and there are fewer motions. New methods are being developed to allow models to be built from such regions of density [56]. In X-ray structures, the map quality is often more uniform than in cryo-EM structures, although flexible loops will also not be resolved. However, loops that are involved in crystal contacts may be defined by clear densities, although care must be taken in their interpretation, as discussed in the following section.

## User beware: crystal contacts

Every structure has caveats, which influences deductions that can be drawn from the structure and the inferences made on the behaviour of the protein *in vivo*. It is reassuring to know that structures of the same GPCR determined by different groups using different techniques are largely identical (within experimental error and when the resolution has been sufficient for confident modelling). It is also gratifying that this holds true for a cryo-EM structure compared with a crystal structure [57]. However, there are always minor differences and, because the activity of a receptor can be affected by subtle changes in, for example, an extracellular loop, then if differences are observed between structures, the question arises whether these have arisen as a consequence of the technique used to obtain the structures or whether they are of biological significance. Below is a selection of observations that we have made in our own laboratory over the past 15 years.

**Figure 2.**
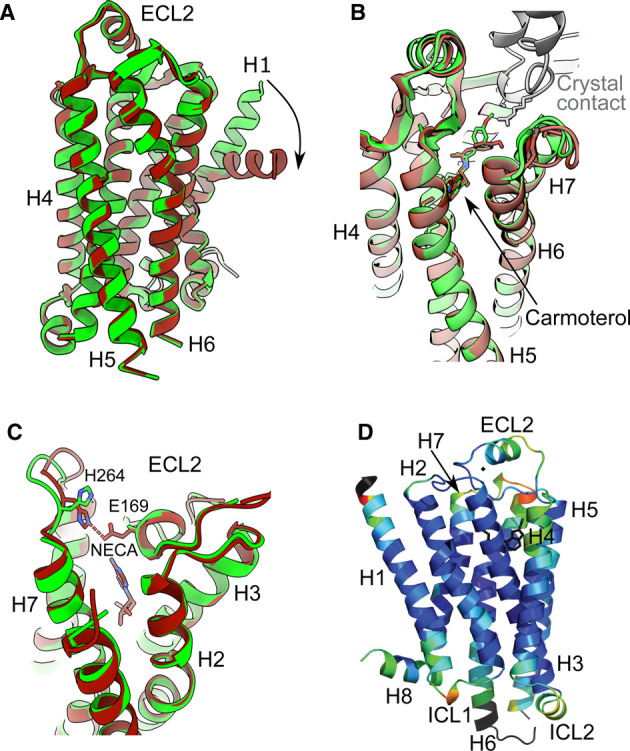
Artefacts in X-ray crystallography. (**A**) 60° kink of H1 in chain A (brown) of β_1_AR compared with chain B (green); PDB ID 2VT4 [23]. (**B**) Different pose of the methylphenoxy moiety of carmoterol in chain A (brown) compared with chain B (green); PDB ID 2Y02 [59]. (**C**) The salt bridge in the extracellular region of A_2A_R determined in the crystal structure at pH 4.8 (PDB ID 5G53) [32] is absent from the cryo-EM structure determined at pH 7.5 (PDB ID 6GDG) [57]. (**D**) The RMSD differences (rainbow colouration) between β_1_AR crystallised in detergent (PDB ID 2VT4) compared with β_1_AR crystallised in lipidic cubic phase (PDB ID 4BVN) were determined and plotted on the structure (red, large differences; dark blue, no differences) [58]. Panel 2D has been reproduced from [58].

The conformation of loops in crystal structures can be affected by the environment and crystal contacts. This is demonstrated by the crystallisation of β_1_AR in detergent or in lipidic cubic phase where the same ligand, truncations and deletions were present in both constructs; differences were greatest in the loop regions and the ends of the transmembrane regions, whilst the core of the receptors were virtually identical (Figure 2D) [58]. Crystal contacts can be very strong and can cause a transmembrane helix to kink through 60° as seen when comparing chain A and B in the β_1_AR structure (PDB ID 2vt4, Figure 2A). In the case of carmoterol-bound β_1_AR, crystal contacts at the top of H7 in chain A forced a change in ligand binding pose, causing it to shift slightly, resulting in a different pose of the methylphenoxy group of carmoterol in comparison with chain B where the crystal contact is absent (Figure 2B) [59]. Crystallisation may also only occur at non-physiological pH, as was observed for the X-ray structure of A_2A_R-mini-G_s_[32] which crystallised at pH 4.8. A salt bridge was observed between His264 and E169 in the entrance to the orthosteric binding site, which could be important in ligand binding (Figure 2C) [60]. However, in the cryo-EM structure determined at pH 7.5, the salt bridge was absent as the respective residues were further apart and adopted different rotamers. Of course, in cryo-EM structures there are no crystal contacts, which avoids the potential pitfalls observed in X-ray structures.

## Access to conformational dynamics of structures

A significant advantage of cryo-EM over X-ray crystallography is the ability to determine structures of flexible complexes. This has been demonstrated with the structure determination of a ‘megacomplex’ formed between G_s_-coupled β_2_AR with β-arrestin bound to the C-terminus of the receptor, and stabilised through the binding of three different antibodies [61]. The complex was very flexible and initially the overall resolution was only 7 Å. However, the separate complexes within the whole structure were masked and individually refined to 3.8–4 Å resolution, and subsequently rigid-body fitted into the 7 Å map. This is not possible in X-ray crystallography.

Protein particles vitrified in solution on a cryo-EM grid preserves the conformational space sampled before freezing. This means that it is possible to access structural information about the conformational landscape of the protein, which for GPCRs is highly relevant given their dynamic nature and wide conformational landscape [62,63]. In contrast, X-ray crystallography can, at best, generate a series of snapshots of a receptor in different conformations, provided that each state can be suitably stabilised and crystallised using, for example, different ligands or antibodies. In cryo-EM, 3-dimensional classification based on maximum likelihood is able to distinguish different discrete conformations occurring in a single dataset of a protein. For example, the neurotensin receptor NTSR1 coupled to G_i_ displayed two different conformations [64], the canonical conformation similar to other GPCR-G_o/i_ complexes, and a less abundant non-canonical conformation. The G_i_ heterotrimer in the latter structure had a more rigid nucleotide-binding domain and the receptor adopted features of both active and inactive states, suggesting this conformation was on the activation pathway to form the non-canonical conformation. Differentially populated conformations have also been described for the mGluA2 structure [65].

Aside from discrete conformations, recent computational advances have allowed the analysis of continuous motion within receptors. Systematic variability analysis performed on cryo-EM datasets applies 3-dimensional probabilistic principal component analyses (PPCA) to the data [66]. This describes a continuum of movements within a cryo-EM dataset and has been performed on GPCR-G protein complexes of the cannabinoid-1 receptor [66], the adrenomedullin receptor [67], the secretin receptor [68] and rhodopsin [69]. The motions present within the different complexes appear to be similar, providing structural information on transient states that might be important to the activation mechanism. X-ray free electrons lasers have also been used to extract dynamic information of GPCRs [70,71], but there is always the possibility that lattice contacts impose restrictions on receptor mobility.

## New biology from cryo-EM structures of GPCRs

An interesting consequence of using cryo-EM to solve GPCR structures has been the discovery of unexpected biology. This has arisen through the ability to express small amounts of protein in insect or mammalian cells and not having to extensively engineer the receptor. For example, the structure of the yeast mating factor receptor Ste2 was determined from the wild type protein, where unexpectedly the N-terminus of the receptor that was predicted to be unstructured was important in forming a domain-swapped dimer interface [38]. In another example, the GPR97-G_o_ complex was expressed and purified from insect cells, and the cryo-EM structure allowed identification of [72] palmitoylation at Cys351 in the G protein α-subunit that was essential for G protein coupling.

There are also cases where insights into lipids have been made possible through the use of mild detergents in the preparation of receptors for cryo-EM. For example, the lipid phosphatidylinositol 4-phosphate was observed at the interface between the serotonin 5HT_1A_ receptor and G_i_, which was shown subsequently to be an important factor in G_i_ coupling [73]. Lipids are also frequently observed in X-ray structures, but very often these are of mono-olein and cholesterol which are present in large excess during lipid cubic phase crystallisation experiments. Cholesteryl hemisuccinate (CHS) is also often added to detergents during GPCR purification to improve receptor stability and CHS will therefore be present upon structure determination by cryo-EM. Caution must, therefore, be given to the assignment of lipid densities to cholesterol, for example, in GPCR structures these densities should be assumed to be CHS unless mass spectrometry identifies cholesterol in the purified receptor. The ability of cryo-EM to determine structures of receptors in lipid nanodiscs or in lipid-enriched detergent micelles, will increase our knowledge of the structural role for lipids in GPCR complexes [9,10].

## Future perspectives

X-ray crystallography is a mature technology that has transformed our understanding of the structure and function of GPCRs. In contrast, single-particle cryo-EM has only recently realised its potential in the GPCR field for routinely producing data at sufficient resolution for model building. The ease of preparing samples for cryo-EM compared with X-ray crystallography makes cryo-EM the method of choice for determining structures of GPCRs coupled to G proteins and arrestins. Detergent-solubilised receptors have dominated recent structures, but the increasing focus on the importance of lipids means that more structures will undoubtedly be determined of receptors embedded in a lipid nanodisc, a platform that is not amenable to analysis by X-ray crystallography. Cryo-EM also permits the possibility of time-resolved studies [74], where G proteins and receptors can be mixed and vitrified with a time resolution of 10 msec; this has the potential to identify the pathway of G protein engagement to a receptor.

There is no doubt that cryo-EM will continue to develop, with better microscopes, detectors and software that will make it easier and faster to determine structures of even small proteins [75,76]. This will mean that cryo-EM will compete directly with areas where X-ray crystallography currently has the advantage, namely rapidly producing multiple structures of the same receptor for SBDD. The development of cheaper microscopes will have a significant democratising effect on the field [77], allowing multiple electron microscopes to be available at all research centres in academia and industry, accelerating progress even further. According to theoretical calculations, it should be possible to determine the structure of a 38 kDa protein by single-particle cryo-EM [78]. Given the current rate of advancements in the field, it is only a matter of a few years before structures of the inactive states of small GPCRs can be determined routinely without the requirement for antibodies or other binding partners.

**Table 1:** Advantages and disadvantages of cryo-EM versus X-ray crystallography

	Cryo-EM	X-ray crystallography
Sample	Membrane protein can be in any detergent or nanodisc	Specific detergents required
	Requires µg amounts of protein	Requires mg amounts of protein
	Can tolerate certain degree of sample impurity and heterogeneity	Requires pure and homogenous protein
	No protein engineeing necessary to remove flexible regions, post-translational modification sites or thermostabilisation	Extensive protein engineering required to get well-diffracting crystals
Structure determination	Does not require crystallization	Requires crystallization
	Preferential orientation of the particles may hinder structure determination and/or reduce the resolution	Crystals may not form or be insufficiently ordered to yield a structure
	Can provide lower resolution information	All or nothing result
	Slow collection and processing	High speed in data collection and processing
	Electron microscopes may be in-house, allowing fast data collection. If electron microscopes are not in-house, then access could be slowed considerably.	Requires access to synchrotron sources that are national facilities, which could slow access for crystal screening and data collection
Structure analysis	No crystal contacts present	Crystal contacts might induce structural artefacts
	Molecules sample conformational landscape as in solution	Restricted access to dynamics

## Conclusions

Cryo-EM has rapidly become the method of choice for the determination of GPCR structures coupled to either a G protein or β-arrestin, and in many instances has become almost routine. Single-particle cryo-EM has opened up new avenues of investigation in GPCR structure biology, including the study of transient intermediates, continuous motions and structure determination of flexible complexes. It is only a matter of time before single-particle cryo-EM will allow structure determination of GPCRs in a native state on its own within a lipid bilayer, without requiring protein engineering or binding partners.

## Perspectives

- GPCRs are the largest family of receptors in humans and are also the most targeted family of proteins by drugs. Structural biology has been essential to understand the molecular pharmacology of GPCRs and to provide a foundation for next generation drugs with improved specificity and reduced side effects.
- Cryo-EM is the preferred technique to determine GPCR structures in the active state coupled to a G protein or arrestin. Currently, X-ray crystallography remains the preferred technique for structure determination of GPCRs in the inactive state as they are usually too small for cryo-EM.
- The technology of single-particle cryo-EM is developing rapidly and will within a few years become the preferred technique for structure determination of any state of a GPCR. This is because it will be very fast and cheap, as extensive protein engineering and crystal optimisation are not required.

## Open Access Statement

Open access for this article was enabled by the participation of MRC Laboratory of Molecular Biology in an all-inclusive Read & Publish pilot with Portland Press and the Biochemical Society under a transformative agreement with JISC.

## Abbreviations

A_2A_R: adenosine A_2A_ receptor
β_1_AR: β_1_-adrenoceptor
β_2_AR: β_2_-adrenoceptor
Cryo-EM: cryo-electron microscopy
GPCR: G protein-coupled receptor
SBDD: structure-based drug discovery
